# The Influence of Adverse Childhood Experiences in Pain Management: Mechanisms, Processes, and Trauma-Informed Care

**DOI:** 10.3389/fpain.2022.923866

**Published:** 2022-06-10

**Authors:** Lydia V. Tidmarsh, Richard Harrison, Deepak Ravindran, Samantha L. Matthews, Katherine A. Finlay

**Affiliations:** ^1^School of Psychology and Clinical Language Sciences, University of Reading, Reading, United Kingdom; ^2^Royal Berkshire Hospital, Reading, United Kingdom

**Keywords:** chronic pain, ACEs, pain catastrophizing, fear-avoidance, person-centered care, treatment adherence and completion

## Abstract

Adverse childhood experiences (ACEs) increase the likelihood of reduced physical and psychological health in adulthood. Though understanding and psychological management of traumatic experiences is growing, the empirical exploration of ACEs and physical clinical outcomes remains under-represented and under-explored. This topical review aimed to highlight the role of ACEs in the experience of chronic pain, pain management services and clinical decision making by: (1) providing an overview of the relationship between ACEs and chronic pain; (2) identifying biopsychosocial mechanisms through which ACEs may increase risk of persistent pain; (3) highlighting the impact of ACEs on patient adherence and completion of pain management treatment; and (4) providing practical clinical implications for pain management. Review findings demonstrated that in chronic pain, ACEs are associated with increased pain complications, pain catastrophizing and depression and the combination of these factors further heightens the risk of early treatment attrition. The pervasive detrimental impacts of the COVID-19 pandemic on ACEs and their cyclical effects on pain are discussed in the context of psychological decline during long treatment waitlists. The review highlights how people with pain can be further supported in pain services by maintaining trauma-informed practices and acknowledging the impact of ACEs on chronic pain and detrimental health outcomes. Clinicians who are ACE-informed have the potential to minimize the negative influence of ACEs on treatment outcomes, ultimately optimizing the impact of pain management services.

## Introduction

Chronic pain is one of the leading causes of disability worldwide [Global Burden of Disease Reviews; (1)], impacting 35–52% of the UK population (2). The negative emotional and sensory experience associated with potential or actual tissue damage [IASP; (3)] means that chronic pain is complex and multidimensional. There is growing evidence to suggest early life experience(s) may influence chronic pain occurrence and presentation in adulthood (4). Adverse childhood experiences (ACEs) are defined as potentially traumatic encounters before 18 years of age (5). These may affect a child or adolescent *directly* through physical, psychological, emotional, and/or sexual abuse or neglect by a primary caregiver, or *indirectly via* environmental exposure [e.g., parent psychopathology, exposure to violence against mother, living with substance abuse, and early parent loss from divorce or incarceration; (5)]. As most recent global estimates indicate more than 1 billion 2–17-year-olds are victims of physical, emotional and or sexual abuse worldwide (6), and 49.8% of children are exposed to at least one ACE during their lifetime (7), identifying the long-term implications of ACEs on development and health outcomes is critical.

The prevalence of chronic pain in adults with ACEs is high, with 84% (*N* = 326) reporting at least one ACE (8). By contrast, in the general population it has been estimated that only 61.6% (*N* = 214,157) of people report at least one ACE (9), a rate substantially below that seen in people living with pain. Furthermore, incidence of chronic pain doubles in individuals with ACEs (8.7%) compared to no ACEs [4.6%; (7)]. Together, these statistics emphasize the higher occurrence of childhood adversity in people living with chronic pain. Higher prevalence of ACEs in individuals with chronic conditions typically increases healthcare utilization as a result of poor adult physical and psychological health (10), highlighting the societal health burden associated with ACEs. Figures suggest the total annual costs of lost productivity due to ill health and premature mortality as a result of ACEs have been estimated at US $581 billion in Europe and $748 billion in North America (11). Furthermore, 75% of these costs result from supporting the needs of individuals with as few as two or more ACEs (11), demonstrating the major societal impact of supporting individuals exposed to ACEs. The interaction between ACEs and chronic pain has been minimally explored, but it is widely recognized that chronic pain itself, independent of ACEs, has substantial physical and psychological impacts on multiple life domains, including reduced mobility, poor sleep, fatigue, distress, and unemployment (12, 13). The pervasive detrimental physical, social and psychological outcomes from chronic pain also commonly present in people who have experienced greater childhood adversity (14, 15). Therefore, there is an urgent need to further interrogate the role of ACEs in pain management and/or comorbid health conditions to develop responsive, ACE-informed pain-related clinical care and decision making.

The overall aim of the present review is to highlight the role of ACEs in the experience of chronic pain and the impact that such ACEs may have on pain management services and clinical decision making. Therefore, the objectives are four-fold: (1) to provide an overview of the relationship between ACEs and chronic pain; (2) to identify possible mechanisms through which ACEs may increase risk of persistent pain; (3) to highlight the impact of ACEs on patient adherence and completion of pain management treatment; and (4) in light of this evidence, to provide practical clinical implications for pain management.

## ACEs and Chronic Pain in Adulthood

A growing body of literature demonstrates harmful effects of ACEs exposure on increasing the likelihood of chronic pain (16, 17) and disability (11) in adulthood. Meta-analytic evidence indicates childhood abuse and neglect is associated with greater pain symptoms and pain-related conditions compared to individuals with no ACEs (18) and chronic widespread conditions like fibromyalgia in adulthood (19). More recently, this trend has been further validated, with increased pain severity observed in people with ACEs (20), suggesting not only that pain occurrence is elevated after exposure to ACEs, but also that the disabling intensity of the pain itself may also be increased. Longitudinal evidence also suggests court-validated ACEs (documented, not self-reported) significantly increase the risk of disabling pain symptoms in middle adulthood 30 years later, compared to a demographically matched control group (21). The link between ACEs and pain in adulthood is therefore severe, prolonged and increasingly well-established.

However, whilst a large body of evidence suggests a positive relationship between ACEs and chronic pain in adulthood, some conflicting findings are evident in the literature base, particularly in terms of which ACEs (for example, physical abuse, sexual abuse, or neglect) are associated with specific pain-related diagnoses. A systematic review including eighteen studies with 13,095 participants found that adult fibromyalgia is significantly related to childhood physical and sexual abuse to a greater extent than other ACEs (22). More recently, meta-analytic evidence confirmed the significant association between both physical and sexual abuse and adult fibromyalgia, although physical abuse was more strongly related (19). Physical abuse in childhood also displays an increased risk of back and neck pain in adulthood (23–25), whereas sexual abuse (as yet) does not (25). Additionally, functional impairment due to pain is significantly predicted by maltreatment, whereas sexual abuse more strongly predicts pain intensity (26). Taken together, the discrepancies in the patterns of association between ACEs and pain conditions may be explained by variations in the ACE dimensions; the type of abuse or neglect, and the links between these and pain outcomes in later life. Thus, it is not that there is no relationship between ACEs and pain, rather the type of ACE exposure may predict the form and severity of the pain experience. The ACE-pain research base, however, is currently fragmented, meaning definitive directional conclusions are difficult to validate.

What is clearer, however, is that people with pain and a history of childhood adversity are not only susceptible to greater physical pain, but to vulnerabilities in their psychological wellbeing. A recent umbrella review of nineteen meta-analyses including 559 studies (*N* = 4,089,547) found childhood sexual abuse is strongly associated with elevated rates of clinical depression and anxiety (14). Evidence suggests a 77% increased likelihood of depression in young adulthood in individuals with ACEs (27). Such meta-analytic evidence shows depression and anxiety are significantly associated with chronic pain (28, 29), with higher levels of psychological distress predicting more severe and disabling pain (30). In combination, this evidence suggests that independently, individuals with pain or ACEs display a greater susceptibility to poor psychological health. Therefore, the presentation of ACEs and pain *in combination* may pose a particularly severe psychological risk profile.

Indeed, comorbid chronic pain and anxiety is related to an increase in suicide attempts (31) and also mediates the relationship between ACEs and greater suicide risk (32). ACEs exposure increases risk of suicidal behavior (33) with 146% greater odds of suicidal ideation in adulthood compared to individuals with no ACEs (27). A recent systematic review, including 28 studies, found ACEs significantly predict suicide attempts and suicidal ideation, with increased likelihood of both ideation and suicide attempts occurring in those with more numerous ACEs (34). This indicates a high-risk profile for individuals with ACEs and pain, demonstrating their increased susceptibility to severe psychological co-morbidities and risks of early mortality. Thus, it is clear that for people living with pain, those with greater ACEs exposure represent an even more psychologically vulnerable subgroup (34).

It is important to recognize that the interrelationship between ACEs and pain cannot simply be explained by the exposure to trauma, and trauma-related biopsychosocial outcomes. While ACEs are potentially traumatic events, trauma may not necessarily be experienced as a result of ACEs. This has been demonstrated through the significant variability in trauma presentation profiles across types of ACEs; sexual abuse is most strongly predictive of trauma (35), whereas parental divorce may not be experienced as traumatic (36). Protective factors such as social support from a nurturing adult (37), living in a safe community (38), and resilience (39) play a protective role in the development of trauma following adversity. Whilst trauma is not *always* a consequence of ACEs, Post-Traumatic Stress Disorder (PTSD) [the intrusive re-experience of traumatic events characterized by hyperarousal and avoidance of trauma-related stimuli; (40)], can often result (41), and there is greater associative risk of PTSD presentation from cumulative ACEs exposure (42, 43). However, research suggests that post-traumatic stress disorder (PTSD), in isolation, is not the unique mechanism by which ACEs facilitate pain; significantly greater pain complications are evident where ACEs and PTSD combine, compared to the (lower) levels of pain typically experienced by people who have been diagnosed with PTSD alone (21). Furthermore, PTSD is *not* found to mediate the relationship between increased pain intensity and ACEs in chronic pain patients (20). It is clear therefore that PTSD must be considered separately from ACEs, and though it undoubtedly affects the pain experience, it is the particular salience of childhood adversity and the subsequent trauma-pain relationship which has a highly problematic impact on pain outcomes.

## ACEs: The “Dose-Response” Relationship

Critical insights on the impact of trauma on chronic health conditions over the life course come from the understanding of the dose-response relationship (44). Trauma has a cumulative lifetime burden, with significant increased risk for poor general health, elevated morbidity (45) and pre-mature mortality (5) observed with greater exposure to ACEs. This powerful cumulative influence of ACEs on adult health is an important predictive factor for subsequent health outcomes. Felitti et al. (5) in their seminal ACEs study, found that within a representative US population sample of *N* = 13,494, those with four or more ACEs had an increased risk for various health conditions including (but not limited to) ischemic heart disease, cancer, obesity, alcoholism, lung disease, and depression. Recent evidence supports this; more numerous ACEs are associated with increased pain intensity, pain interference, anxiety, and depression (8, 16, 18). Furthermore, as the number of ACEs increase, the higher the level of associated psychosocial functioning impairment observed (46). Indeed, greater ACEs exposure (≥3 ACEs), in a “dose-response” relationship, is associated with a higher level of clinical complexity in terms of adjustment to pain and mental health symptoms (47, 48). This therefore suggests a greater vulnerability to poor health outcomes for individuals with higher exposure to childhood adversity. By identifying individuals with higher numbers of adverse childhood experiences, it may be possible to offer additional support to facilitate improved responsivity to pain management interventions.

While evidence suggests a “dose-response” relationship between multiplicative ACEs and worsened pain-related health outcomes (8), response to treatment does not appear to follow the same trajectory. Following pain rehabilitation, significant improvements in pain, physical and psychosocial functioning were observed in all groups (0 ACEs, 1–2 ACEs, or ≥3 ACEs), irrespective of the number of ACEs (47). This suggests that while individuals with greater ACEs exposure may be more susceptible to chronic pain and clinical complexity in adulthood, response to treatment is equally promising in those with and without childhood adversity. Investment in pain management services is therefore key as it may provide an opportunity to mitigate the ACEs-pain relationship. This poses an important point for further investigation: it is yet to be understood how the mechanisms underlying the ACEs-pain relationship in adulthood differentially influence the pain experience and response to treatment.

While the dose-response relationship suggests a cumulative effect of ACEs on negative health outcomes (5, 44), the influence of age of exposure remains understudied. Most rapid neuroendocrine development occurs from birth to 6 years, therefore implicating early childhood as a sensitive period during which the detrimental impacts of ACEs may be heightened (49). Brain development, endocrine and immune system physiology can be dysregulated by prolonged and unrelenting exposure to stress(ors) (50). Indeed, ACEs in early childhood lead to increased rates of adult PTSD and internalizing symptoms (51, 52), more severe depression, anxiety, and suicide attempts (53, 54). As elevated rates of anxiety and depression are associated with ACEs (14), chronic pain (28, 29), and more severe and disabling pain (30), together, this may suggest individuals exposed to ACEs in early childhood potentially have an increased risk of pain intensity and psychopathology. However, significant maturation of neuroendocrine development also occurs during adolescence (55). Adolescence is also characterized as high risk for psychopathology onset due to shifts in biological, cognitive and social systems (56). This therefore potentially poses ACEs exposure during this time as a highly sensitive period to psychological distress. Indeed, adolescents with cumulative ACEs exposure are two times more likely to develop depression, anxiety and suicidal ideation (57). Similarly, in terms of chronic pain conditions (without specific comorbid mental health difficulties), higher numbers of ACEs are associated with fibromyalgia and recurrent headache in adolescents (58).

Though the research in ACE dose-responsiveness is fledgling, it is important to note that while developmental patterns may exist, current assessment measures do not differentiate by the age at which the ACE occurred. A recent umbrella review of systematic reviews and meta-analyses concluded the effect of age of exposure on mental health could not be explored due to an insufficient database (59). Moreover, there is no direct research on the direct impact of age of ACEs exposure on subsequent diagnosis of chronic pain in adulthood. This therefore limits the capacity to finitely conclude what extent the predictive value of age of ACEs has on subsequent pain presentation. Improvements in the way ACEs are measured are required to gain greater insight into the developmental trajectories and mechanisms underlying ACEs and pain.

## Biological Mechanisms and Processes

Psychoneuroimmunology emphasizes the interaction between behavioral, neural and immune processes on health (60). This perspective is aligned with the biopsychosocial approach (61), recognizing the interacting biological, psychological and social factors influencing the development and maintenance of chronic pain. From a psychoneuroimmunological perspective, exposure to adversity during the period of critical neuroendocrine development within childhood, can result in altered stress reactivity, physiological sensitization to stress and immunological dysregulation (62). Longitudinal systematic and meta-analytic review evidence suggests that dysregulation of the stress and immune systems as a result of ACEs exposure, is significantly associated with elevated inflammatory biomarkers (63, 64), demonstrating a mechanistic physiological response to trauma. This prolonged inflammation can cause peripheral sensitization, resulting in hyperalgesia and chronic widespread pain (65). Stress and immune system dysregulation persists into adulthood, associated with various pain somatization conditions including fibromyalgia and chronic pelvic pain (19, 66). Additionally, more severe inflammatory effects are found in people with cumulative ACEs exposure, even up to 30 years later (67, 68). Typical confounding factors, such as low socioeconomic status, smoking or diet, had no impact on this relationship (68), despite their documented detrimental impact on the immune system and resulting increased inflammatory activity (69). This demonstrates the particular salience of ACEs exposure on the physiological stress response—it is impactful above and beyond poor health behaviors, socioeconomic and environmental risk factors. Thus, understanding the interaction between behavioral, neural, and immune processes on the development of chronic pain in adulthood is important to shape trauma-informed care for pain management.

Allostasis is the process governing the long-term physiological responses to the psychological stresses of both everyday life and major life events (70). Typically, during periods of perceived stress, cortisol is released within the Hypothalamus-Pituitary-Adrenal (HPA) axis, enhancing threat detection and activating the immune system (71). Elevated cortisol levels signal the occurrence of an adequate stress response. When these signals are detected, the body subsequently stops producing cortisol and levels dissipate (72). However, at the point where sustained environmental challenge exceeds coping ability, like when a child faces adversity, this process can become chronically activated and no longer adaptive (73). This results in allostatic overload (74) which occurs *via* three dominating pathways: firstly, through frequent and repeated exposure to a perceived stressor; secondly when the stress response continues following the end of a stressful episode; and thirdly, when the stress response becomes maladaptive and the complex hormonal control system is disrupted (75). Allostatic overload during childhood can lead to epigenetic modulation of this stress response system (76) which increases the reactivity of the HPA axis. This ultimately results in dysregulated cortisol and chronic low-grade activation of the immune system (73). Cortisol is importantly implicated in threat perception (77). Further, the amygdala regulates the perception of threat and the conditioning of the stress response by promoting glucocorticoid secretion, activating the HPA axis (78). Indeed, the association between cortisol and elevated amygdala activity during fearful and anxious states is well-established (77, 79–83). When a stimulus is interpreted as a potential threat in the amygdala, cortisol secretion, which in turn, strengthens synaptic connectivity and promotes dendritic growth in the amygdala, promotes the formation of fear-based memories in animals (77, 79). This mechanism is supported within humans, with a systematic review including 48 fMRI studies demonstrating that the amygdala was the most consistently activated brain region within fear-conditioning experiments (80). Fear-based memory formation can also contribute to conditioning a sensitized stress response, further dysregulating HPA axis activation (84). Chronic HPA dysregulation is observed in individuals with ACEs as indicated by hair cortisol levels (85), and meta-analytic evidence suggests significantly elevated levels of the inflammatory markers peripheral C-reactive protein (CRP), tumor necrosis factor alpha (TNF-α), and interleukin 6 (IL6) in trauma-exposed individuals in adulthood (63). Inflammatory markers appear to differ depending on type of ACEs; physical and sexual abuse is related to a significant increase in TNF-α and IL-6, and parental absence is primarily associated with elevated CRP levels (63). In animal studies, rodents exposed to maternal separation display altered inflammatory profiles, increased pain sensitivity and anxiety (86–88). Such inflammatory biomarkers result in changes in the immune system, ultimately increasing susceptibility to illness and disease later in life (69). Indeed, chronic stress-induced HPA reactivity is also consistently linked to pain conditions including fibromyalgia, chronic pelvic pain, and musculoskeletal pain (66, 89, 90). Thus, HPA axis dysregulation and inflammatory pathways influenced by ACEs exposure can have persisting health implications in adulthood. Further research is needed to explore differential inflammatory effects across type of ACEs exposure.

Neuroinflammation, characterized by activation of microglia and astrocytes, is also increasingly recognized as an important underlying mechanism in the pathogenesis of chronic pain (65, 79, 91, 92), and is observed in conditions like fibromyalgia (93). Microglia, a specialized macrophage with receptors, functions to protect the CNS (94). They do this by integrating and managing the response to glucocorticoids including cortisol and releasing inflammatory mediators such as interleukin-1 and TNFα, alongside catecholamines like noradrenaline (95). In chronically stress-induced systems, like in those associated with ACEs (10, 63), microglia may become overactivated, increasing production of proinflammatory cytokines (96). It is proposed through this inflammatory mechanism, ACEs-related functional and structural brain alterations are observed in regions responsible for memory and emotion regulation including the amygdala and hippocampus (97). Altered microglia activation is also associated with increased anxiety and depressive symptoms (98), common psychological comorbidities of chronic pain (14, 99). Together, this suggests that early life stress may prime microglia to overrespond and facilitate inflammation, each increasing the likelihood of chronic pain (63, 100, 101). This, alongside epigenetic changes occurring in stress response genes as a result of exposure to ACEs (102), are both mechanistic avenues warranting further investigation in relation to elevated threat perception in individuals with ACEs [pain catastrophizing; (103)]. As elevated catastrophizing and threat perception is also associated with increased pain intensity (88), this would help to support subgroups at increased susceptibility to worse pain. Despite the advances in the psychoneuroimmunological understanding of ACEs, it is important to note that much evidence is based upon animal experimentation (104), limiting its potential extrapolation to human models. Concepts such as the role of cortical modulation or human psychology are not yet elucidated, and further investigation is required.

In summary, allostatic overload occurs when environmental stressors exceed an individual's ability to cope. This disrupts the stress response, affecting endocrine, neural, and immune systems. When chronically activated, as in individuals with ACEs, the physiological stress response becomes hypervigilant in detecting threat, increasing pain perception. These biological mechanisms within the stress response and inflammatory biomarkers therefore provide potential targets for intervention for stress management for clinical subgroups with increased susceptibility to chronic pain.

## Psychological Theoretical Models

Pain-related research evidence and pain theory agrees that biological mechanisms alone cannot adequately explain processes or mechanisms underlying pain (105–108). Rather, an integrative biopsychosocial perspective is optimal, acknowledging the importance of how interacting psychological and social factors may influence the experience of pain (109). Moreover, psychological models and theory offer valuable understanding and insight to intervention in order to support behavior change (110). Thus, exploring these factors in understanding the processes underlying the ACEs-pain relationship is key to informing clinical practice. There are many cognitive and emotional factors that are valuable when considering the clinical implications of ACEs within pain management, particularly the concepts of pain catastrophizing and fear-avoidance, and their role in the ACEs-pain relationship. As demonstrated, there is an empirically grounded link between ACEs and threat detection through stress-induced HPA axis dysregulation, increasing amygdala activity and fear-based memory formation (77, 79–83). Moreover, the link between heightened threat detection and chronic pain is also well-established (66, 89, 90). Based on these biological mechanisms, psychological models of threat detection are likely to be relevant in further understanding how ACEs may influence pain.

## Cognitive Factors: Pain Catastrophizing

Cognitive-Behavioral models (111) emphasize the salience of cognitive and behavioral factors in the maintenance of chronic pain. Like with other aversive life events, cognitive factors include primary and secondary appraisal of the severity of the perceived threat and the ability to overcome the stressor (112). Within the context of chronic pain, the sheer pervasiveness of pain can be very distressing, which facilitates heightened negative appraisal, in turn making the experience of pain worse (111). Pain catastrophizing (PC) is a concept characterized by such negatively oriented cognitive appraisals; the tendency to magnify the threat and interpretation of pain (113). PC is comprised of three dimensions: Rumination, repetitive focus on symptoms of distress; Magnification, an exaggerated perception and anticipation of the threat of pain; and Helplessness, a perceived inability to exert control (114–116). Evidence from two meta-analyses concludes PC is the strongest psychosocial predictor of chronic pain following surgery (117, 118). Therefore, when adopting a biopsychosocial approach to better understand the development and maintenance of chronic pain, the salience of catastrophizing is evident. Threat salience models explain that threat learning, with pain perceived as a cognitive-emotionally salient threat, mediates the link between stress and chronic pain (119). Thus, following exposure to ACEs, where there is a prolonged and hypervigilant stress system dysregulation (63, 69, 85), PC may act as a cognitive mechanism contributing to this stress response. Indeed, pain magnification, rumination and helplessness dimensions within PC are found to increase cortisol secretion (113, 120, 121). Given the link between chronic stress-induced HPA dysregulation and pain (66, 89), this may be a cognitive-emotional mechanism through which individuals with ACEs display an increased risk of chronic pain likelihood and pain intensity (7, 20). Further mechanistic research is required to fully elucidate the link between ACEs, threat perception and chronic pain in adulthood. Nonetheless, this evidence highlights that people with ACEs are an especially susceptible population at risk of more severe chronic pain, potentially mediated *via* PC mechanisms.

Indeed, in the context of ACEs, individuals with a history of childhood maltreatment display a heightened perception of threat and increased anxiety as a result of their early trauma exposure (122). This is associated with an increased likelihood of PC in people with ACEs (103) compared to individuals with no ACEs (123). Moreover, this association is evident in adults with few ACEs (123), even when controlling for confounding factors like socioeconomic status, depression and anxiety (103). Importantly, evidence focusing on change processes during treatment found reductions in PC are central to successful Pain Management Programme (PMP) outcomes (124, 125). Together, these findings emphasize the salience of ACEs on PC in adulthood, and the negative implications of this mechanism on treatment outcomes. In chronic pain patients, a history of ACEs is associated with greater PC and a more severe clinical profile, characterized by higher levels of pain interference and pain severity (126, 127). Collectively, the evidence suggests that assessing a patient's history of ACEs could facilitate appropriate intervention for this vulnerable population to overcome maladaptive coping and poor pain outcomes.

In summary, PC exacerbates pain *via* a heightened perception of threat in individuals with ACEs (122). This is particularly salient in the context of ACEs as it is the strongest psychosocial predictor of chronic pain (118) and reductions in PC are central to change processes within PMP treatment (124). Thus, an increased susceptibility to pain catastrophizing in ACEs pain patients (103) highlights the importance of trauma-informed care for pain management. Trauma-awareness in assessment and formulation can therefore help to support emotional and behavioral factors relevant to pain self-management.

## Behavioral Factors: Pain Fear-Avoidance and Trauma Experiential-Avoidance

Evidence suggests that PC precedes pain-related fear (128). The fear-avoidance model (129) explains potential pathways in which pain patients enter a downward spiral increasing and maintaining avoidance behavior, pain, and disability. Fear is an emotional response to anticipated threat. Fear of pain encompasses beliefs and expectations regarding the extent of detrimental impact that pain will cause (130). When pain is cognitively magnified and perceived as an imminent threat, as with catastrophising, avoidance is elicited as a protective fear response to prevent an encounter with or escape from the pain-related stimulus (129). Avoidant behavior often results in activity restriction (expected to cause pain), interfering with the ability to engage in valued life experiences, and increasing the risk of negative mood and functional disability (129). Fear-avoidance behaviors are activated in response to the anticipation of pain, as opposed to the retrospective processing of pain. Therefore, by avoiding activities expected to exacerbate pain, there are limited opportunities to correct invalid/inaccurate perceptions of pain in the context of physical threat. This therefore reinforces catastrophized pain beliefs and avoidant behavior, thus maintaining the cycle of fear-avoidance and chronic pain. Indeed, avoidance behavior is associated with increased pain intensity and difficulties adjusting to pain (131). This suggests fear-avoidance is a central cognitive-behavioral mechanism to persistent pain and offers an important target for intervention. These observations are even more salient in the context of long waiting lists for pain management/treatment, characterized by uncertainty (132) and elevated anxiety (133), as pain fear-avoidance is likely to also be exacerbated given the central role of anticipation. Therefore, in order to improve treatment engagement and outcomes, further study on improving pain fear-avoidance during this period is required.

Fear-avoidance is particularly important in the context of ACEs and traumatic experience. In trauma-exposed individuals, avoidance of trauma-related stimuli is observed as a protective affective-behavioral response. This is a dominant characterization of PTSD, termed experiential-avoidance (40). Experiential-avoidance, similar to fear-avoidance, is an inflexible emotional regulation strategy characterized by exerting effort to avoid unpleasant emotions, cognitions, memories and internal experience (134). While it is important to recognize that PTSD and ACEs are separate constructs, they commonly present together as comorbid and, often, display a shared experience of trauma (41), thus highlighting their association. Neurological markers also indicate ACEs and PTSD are related (135). Hyperactivity in the amygdala, due to its prominent role in threat detection (136), is observed in both PTSD (137) and in people with ACEs, even in those who do not have comorbid mental disorders (138). It is likely therefore that fear-avoidance in pain and experiential-avoidance in trauma may display a mechanistic overlap underlying the ACEs and pain relationship. Evidence supports this as experiential-avoidance and pain fear-avoidance are directly associated in chronic pain populations (139). Moreover, higher post-traumatic stress symptoms, including experiential-avoidance, influence the association between childhood maltreatment and increased pain intensity and more widespread pain in adulthood (140). Within experiential-avoidance, when chronic pain patients with a history of trauma attempt to control negative emotion, this leads to greater trauma-related symptoms, higher pain intensity, pain disability and difficulty coping with pain (99, 139, 141, 142). Higher levels of experiential-avoidance in chronic pain patients are also associated with increased depression and anxiety (143). Moreover, higher levels of avoidance are significantly related to lower completion of psychological intervention in youth with a history of sexual abuse (144). Together, the shared emotional-behavioral mechanisms of fear and experiential avoidance within both ACEs and chronic pain, facilitate increased vulnerability to pain-related outcomes and treatment attrition. As the inability to control negative emotion impacts various dimensions of pain (99), this highlights the critical importance of developing standardized trauma-informed care for ACEs pain patients. To elucidate the mechanisms underlying ACEs and pain more fully, future research would benefit from exploring the overlapping constructs of fear and experiential avoidance and how they work within the ACEs and pain relationship.

## The Impact of ACEs on Patient Adherence and Completion

Longitudinal evidence indicates a history of childhood neglect or sexual abuse predicts early attrition in psychotherapy in adulthood (145). Childhood sexual abuse also increases risk of treatment non-adherence and non-completion in a variety of interventions (146–148). In a low-income sample in Australia, a history of abuse alone significantly predicted early attrition of a pain self-management programme (149). Furthermore, elevated pain catastrophizing and substance use history, additional factors commonly associated with ACEs (103, 150) also predicted non-completion of treatment (149). This suggests people with ACEs display a higher risk of attrition or early withdrawal from treatment interventions. To date, there has been no literature exploring the impact of ACEs on patient adherence or completion specific, therefore, further research is recommended for greater understanding of how ACEs-pain patients can be supported in order to increase PMP engagement and completion.

## The Impact of COVID-19 on ACEs and Pain

For chronic pain patients, COVID-19 has exacerbated pain symptoms as a result of pervasive social disruptions, reduced access to or discontinued treatment over health concerns (151, 152). This will likely lead to an increased demand on healthcare services as a result of treatment backlogs and worsening pain conditions. However, COVID-19 has not only impacted healthcare service pressures, it has also caused significant psychosocial disruptions in the home. Parental stress, depression, financial burden, and substance abuse are all risk factors for child abuse and maltreatment (153). Each of these factors have been exacerbated by the socioeconomic and psychological costs of the COVID-19 pandemic (154). Furthermore, social distancing, stay at home orders and the removal of social support has had detrimental impacts on individuals living in adverse environments (155). While real-time data on the effects of COVID-19 on ACEs is scarce, reports indicate increasing incidents of ACEs. From April-September 2020, children suffering serious harm or death from abuse or neglect increased by 25% [(156); Childhood Safeguarding Practice Review]. Recent findings also show that during the pandemic, children who reported ACEs reported a 29.1% increase in witnessing domestic abuse and 42.2% increase in emotional abuse (154). Latest figures from the Office of National Statistics show a 7% increase of domestic abuse-related police recorded offenses of 259,324 from March to June 2020 (the height of lockdown restriction in the UK) compared to the same period in 2019, and a 18% increase from 2018. Moreover, demand for domestic abuse helplines increased by 22% from March 2020 to March 2021, suggesting an increased occurrence of abuse and reduced access to external support services (157). As trends prior to the pandemic suggest a year-on-year average of 6% increase in domestic abuse (158), these figures therefore display notably elevated levels. Furthermore, as enforced isolation with abuse perpetrators can reduce disclosure to services (159), these numbers may continue to rise over the next year as lockdown measures have been lifted for some time, giving an even greater insight into the effect of the pandemic on ACEs exposure. As the impact of the pandemic continues 2 years on, together this evidence suggests the exposure of ACEs may be prolonged, and the significance on resulting health outcomes increased, due to the contextual shifts from the enforced isolation. When combined with the well-established longitudinal evidence of ACEs and poor health outcomes as previously discussed (14), this warrants concern for the long-term impacts of COVID-19 in the coming years. Thus, it is imperative to further understand this relationship in chronic pain patients as both cases and service pressures are anticipated to grow.

Prior to the COVID-19 pandemic, individuals with ACEs already display an increased vulnerability to depression (160), however this is more pronounced in the current climate (161). Recent findings suggest individuals with more ACEs experienced heightened risk perception of COVID-19 and greater depressive symptoms as a result (161). As patients with depression have higher non-completion rates in PMPs (162), this emphasizes how people with pain who also present with ACEs may be a vulnerable and disadvantaged population, risking poorer health outcomes. Furthermore, this also highlights the salience of depression in patient adherence to pain treatment. Persistent victimization, learned helplessness, and negative appraisal, all characterizing depressive symptoms (163), play a role in increased perception of COVID-19 risk and depression. Moreover, it is likely that if an individual with ACEs were required to shield during the pandemic due to their pain-related condition and/or comorbidities, the ACEs may compound this due to an inability to avoid negative home life, exacerbating depression, pain, and access to treatment. Thus, further investigation into how treatment engagement can be optimized for patients with ACEs is important, particularly as the implications of COVID-19 continue.

Depression and psychological distress as a result of the pandemic not only has unequivocal impacts for individuals with ACEs on their personal health, but intergenerational effects are also observed. For example, recent studies show that during the pandemic, parents with higher numbers of ACEs display greater levels of negative parenting and associated child behavioral problems (164, 165). This may suggest the adversity of the current climate may have consequential impacts on the intergenerational transmission of trauma through negative parenting. Increased ACEs in children of this generation may therefore result, exaggerating a cycle of ACEs and poor health outcomes in years to come. Indeed, evidence suggests a relationship between greater parental ACEs and offspring chronic pain (166) and increased child depressive symptoms (8). In a sample of 170 parents of youths with chronic pain, 68% reported at least one ACE and 24% reported four ACEs or more (166). Together, this evidence demonstrates how parental ACEs can influence child pain; depression; and behavioral problems, increasing the likelihood of later abusive or aggressive behavior in adulthood (167–169). Moreover, this suggests each of these outcomes (child pain, depression and behavioral problems) has potential intergenerational effects on both pain and ACEs.

## The Role of ACEs in Waiting for Pain Management Treatment

Regarding clinical decision-making for facilitating effective pain management, it is essential to consider waiting times and the associated declines. Current clinical practice cannot be formulated or implemented without considering the pervasive societal impact of the COVID-19 pandemic, which has further increased healthcare service pressures. Extensive Pain Management Programme (PMP) waitlists have ensued as a result, adding increased pain duration, permanent disability benefits and rates of unemployment to the pre-existing burdens of living with chronic pain (170, 171). Critically, significant declines in psychological wellbeing (172), elevated anxiety and depression are also observed during long waitlists (133). This psychological decline, pervasiveness of persistent pain and feelings of helplessness during long treatment delays (173) often leads to emotional fatigue and patient passivity, which in turn, reduces treatment engagement (174). As depression increases PMP attrition rates (162) and levels of depression have been exacerbated by COVID-19 in individuals with ACEs (161), patients with a history of ACEs may specifically experience greater barriers to treatment engagement as a result. Moreover, given that patients with ACEs demonstrate increased vulnerability to suicide and self-harm (32), understanding how this population can be supported during this period of psychological decline is imperative. Together, this further demonstrates the importance of developing trauma-informed care to that of pre-intervention, to protect patients with ACEs and prepare them for pain-management treatment.

The mechanistic evidence regarding the ACEs-pain relationship and the influence on treatment engagement discussed within this review poses points for intervention (including pain catastrophizing and fear/experiential avoidance), shaping trauma-informed care for pain management. Pain catastrophizing, the strongest psychosocial predictor of chronic pain (118), is especially important in the context of long waitlists characterized by uncertainty (132) and elevated anxiety (133). As such, this may represent a bidirectional barrier for chronic pain treatment. Firstly, there is likely an increased risk of pain catastrophizing during this period, but also, the influence of catastrophizing on health outcomes may become more intense and detrimental. Moreover, elevated pain catastrophizing observed in individuals with ACEs (103) also predicts non-completion of treatment (149). Thus, in terms of clinical application, patients with ACEs may require additional support during the waiting list period to improve treatment adherence and completion to avoid increasing inequity for this disadvantaged clinical subgroup.

## Working With ACEs In Pain Management: Clinical Implications for Treatment

The widespread prevalence of ACEs amongst pain patients, as well as the complex relationship between ACEs and pain, means that clinical services need to adapt to support the patient in a holistic, trauma-informed manner. In order to do so, pain services need to move from a disease-focussed model to a more integrative whole person biopsychosocial approach, utilizing trauma-informed care (TIC). TIC recognizes the prevalence and widespread impact of trauma on the life course and health outcomes of a chronic pain patient (175). It applies a biopsychosocial approach in re-building physical, psychological and emotional safety, promoting patient empowerment (176). Thus, when calibrating patient-focused pain self-management, it is critical to take into consideration the influence of biopsychosocial mechanisms and how they may facilitate improved clinical outcomes and reduce treatment attrition rates. Within pain management, TIC offers a model for development and implementation of preventative approaches (177), shifting the clinical approach from “What is wrong with you” to “What happened to you?” (178). As patients with high ACEs typically utilize higher clinical resources (179), this approach is likely to be valuable for the patient and health-care provider alike. As such, the training of healthcare workforces in TIC is currently being explored in the UK to improve health outcomes for individuals with ACEs (180). In reality, this will require a cultural shift within healthcare to ensure system level changes are put in place to support individuals with ACEs. Thus, raising awareness of the links, mechanisms and processes increasing susceptibility of people with ACEs to worse pain and related conditions is important to support this transition.

Individuals with ACEs present an increased susceptibility to pain complications and barriers to treatment engagement (179). Therefore, the utilization of preclinical ACEs screening would facilitate the early identification of critically vulnerable patients, potentially allowing for stratification of treatment and the formulation of trauma-informed pathways. Screening for ACEs in primary care is common and has not been linked to any added demand for other services (181–183). Rather, an ACE informed approach can reduce subsequent physician visits (184). In the context of long waiting lists, ACEs assessment facilitates trauma-informed care to extend to the pre-treatment period, activating such critical time windows, to prepare clinically vulnerable patients for pain management.

For the purposes of preclinical screening, the most commonly used ACEs conceptualization and scale is the ACE Study Questionnaire [ASQ; (5)]. This is a 10-item scale assessing physical, psychological and sexual abuse; parent psychopathology; exposure to violence against mother; living with substance abusers and early parent loss from divorce or incarceration. The scale is a valid and reliable measure of ACEs, that is stable and robust even within the context of varying health state and symptoms of depression (185). The ASQ also has good convergent validity as shown by significant correlations between the ACEs scale and the other measures of stressful events and trauma (SLEQ: τ = 0.29, *p* = 0.003, CTQ: τ = 0.47, *p* < 0.001) (186). Therefore, the ASQ is a suitable measure for pre-clinical screening, however for ethical considerations, it is recommended a trigger warning is used to prepare patients for potentially distressing content.

The biopsychosocial mechanisms outlined in this review highlight the importance of understanding the role of ACEs in changing the experience of pain, and the application of trauma-informed approaches for person-centered clinical assessment and care provision. The NICE guidelines for chronic pain treatment (187) suggest that it is paramount that individual patients are supported in exploring how their personal life experiences may contribute to their pain. Raising clinician awareness of the relationship between ACEs and pain, alongside recognizing and managing the interplay between ACEs and other comorbid physical and psychological conditions, would optimize person-centered assessment. This increase in awareness could directly facilitate the provision of more relevant information for the patient which may have gone otherwise undetected. The physical and psychological comorbidities associated with ACEs include functional disability, clinical depression and anxiety (14, 99), which further impair the ability to engage in a fulfilled life. By reviewing and managing such interacting factors, an ACE-informed clinician could establish precursors/maintaining factors involved the pain experience and ultimately support the delivery of person-centered care (187).

Both person-centered and trauma-informed care approaches emphasize the importance of active listening and a compassionate approach, with specific focus on the first consultation for building the therapeutic alliance (188, 189). In this context, the therapeutic alliance must involve mutual trust, agreement on goals and methods of reaching them (190) and has been shown to significantly and reliably influence treatment outcomes (191). The therapeutic alliance is particularly important within trauma-informed care as empowerment, patient choice, collaboration and trustworthiness are central tenets of the approach (178). This is especially important for enabling people who have been exposed to ACEs to increase their autonomy, which, through abuse and maltreatment, may have been lost (189). Indeed, when engaging with people with ACEs, simply asking open questions and actively listening to their disclosures of individual experiences has positive effects on health (192). This demonstrates that not only is effective communication required for building a strong therapeutic alliance, but also that this communication can have positive impacts on health directly. Whilst recognizing that embracing a trauma-informed approach is important, practicing within one's scope of practice is also imperative for patient safety (193). Therefore, some associative psychological comorbidities such as PTSD may fall outside the remit of referral for somatic conditions and referral to appropriate practitioners may be required.

## Conclusion

The pandemic has emphasized the importance of amalgamating multiple evidence-bases and adapting to the needs of vulnerable populations, for the protection of physical and psychological health. Through the biological, psychological and behavioral mechanisms explained in this review, ACEs are associated with greater pain intensity, pain interference, likelihood of chronic pain conditions, pain catastrophizing and depression (56). Not only do ACEs alone predict non-completion of treatment (149), pain catastrophizing (103), and depression (162), combining these factors together further increases the risk of treatment non-adherence and early attrition. Therefore, the acknowledgment of ACEs in this clinical sample is critical for improving treatment and protecting this vulnerable population. Sadly, ACEs are more prevalent than ever, due to the isolation experienced during the pandemic and the pervasive associated psychological impacts (154). Moreover, when combined with the typical psychological decline during the waiting list period (172), and service backlogs as a result of COVID-19, the need to retain patients in treatment is an ever-increasing issue. Clinically, patients can be supported through a trauma-informed workforce, employing ACEs awareness approaches and interventions which acknowledge the patient experience to improve pain and health outcomes. However, further study is essential to understand how these populations can be supported during the waitlist period to encourage PMP treatment adherence and completion.

Childhood ACEs have long been identified as a source of persistent vulnerability (4), and the influence of ACEs needs to be prioritized in the context of chronic pain. Culturally, understanding of and empathy toward traumatic experiences is increasing, however, the empirical exploration of ACEs and clinical outcomes remains under-represented and under-explored. Chronic pain is a challenging condition to manage, and can pose a serious impediment to living a fulfilled life (129). It is critical that the knowledge base on ACEs and trauma-informed care is broadened to chronic pain. Further research on how to identify, manage and treat people living with pain after ACEs requires extensive study, especially within the perspective of the impact of COVID-19 and lockdowns. Ultimately, by ensuring that clinicians and healthcare professionals working in pain management are ACE-informed, there is increased likelihood that the negative influence of ACEs on treatment outcomes can be minimized and potentially even redressed, optimizing the impact of pain management services.

## Author Contributions

SM: biological mechanism section. DR: clinical implications section. RH and KF: mentoring, supervision, and reviewing of manuscript. LT: first author for whole manuscript. All authors contributed to the article and approved the submitted version.

## Funding

Open Access Funding has been approved by the University of Reading.

## Conflict of Interest

The authors declare that the research was conducted in the absence of any commercial or financial relationships that could be construed as a potential conflict of interest.

## Publisher's Note

All claims expressed in this article are solely those of the authors and do not necessarily represent those of their affiliated organizations, or those of the publisher, the editors and the reviewers. Any product that may be evaluated in this article, or claim that may be made by its manufacturer, is not guaranteed or endorsed by the publisher.
